# A high-sensitivity, high-throughput newborn screening assay for congenital cytomegalovirus—is it time for universal screening in the United Kingdom?

**DOI:** 10.3389/fped.2025.1543132

**Published:** 2025-03-18

**Authors:** H. Payne, M. Aaltoranta, V. Veikkolainen, N. Kent, T. Gkouleli, A. Lennon, T. Ramgoolam, S. P. Adams

**Affiliations:** ^1^ Section of Paediatric Infectious Disease, School of Medicine, Imperial College London, London, United Kingdom; ^2^ Research and Development, Revvity Inc., Turku, Finland; ^3^ SIHMDS-Haematology, Great Ormond Street Hospital for Children NHS Foundation Trust, London, United Kingdom; ^4^ Department of Virology, Great Ormond Street Hospital for Children NHS Foundation Trust, London, United Kingdom; ^5^ Department of Infection, Immunity and Inflammation, Institute of Child Health, London, United Kingdom; ^6^ Newborn Screening Unit, Great Ormond Street Hospital for Children NHS Foundation Trust, London, United Kingdom

**Keywords:** congenital cytomegalovirus, infants, neurodevelopmental impairment, hearing loss, delays, universal screening

## Abstract

**Introduction:**

Congenital cytomegalovirus (cCMV) is the leading cause of neurodevelopmental and hearing impairment resulting from *in utero* infection, affecting over a million infants globally each year. Early antiviral treatment can limit sequelae; however, most newborns are diagnosed late—or not at all—due to the lack of universal screening. Ensuring the availability of appropriate screening tools is critical to facilitate accurate and timely cCMV diagnosis.

**Methods:**

A high-sensitivity, high-throughput commercial CMV PCR kit targeting the RRP30 control gene and a conserved region of CMV DNA was provided by Revvity and tested in three population groups: (1) leftover dried blood spot (DBS) samples from the UK newborn screening programme, (2) DBS samples from children with CMV viraemia unrelated to cCMV, and (3) DBS and dried saliva samples from infants with and without cCMV.

**Results:**

Of 3,345 anonymised newborn DBS samples analysed, CMV was detected in 22 cases (0.66%), with a mean cycle threshold value of 36.70 (range 31.87–41.68). Assay development demonstrated a sensitivity of 2.04 CMV IU per reaction. This level of sensitivity was replicated using DBS samples prepared from infant/child blood samples with known levels of CMV, suggesting that the sensitivity reflects 2,000–3,000 CMV IU/mL blood.

**Discussion:**

We demonstrated high analytical sensitivity of the qPCR assay with an optimal extraction protocol, making it an effective strategy for cCMV screening using DBS samples. These data suggest a potential cCMV incidence rate of up to 0.66% in the United Kingdom, equivalent to 3,960 infants per year, 25% of whom may develop long-term sequelae, which could be improved through early diagnosis and treatment.

## Introduction

Cytomegalovirus (CMV) is the leading cause of neurodevelopmental and hearing impairment from a congenital infection globally (1). Evidence from randomised control trials (RCTs) supports early treatment with valganciclovir to reduce hearing loss and improve neurodevelopment outcomes (2, 3). However, despite its prevalence, profound clinical impact, and available treatment, newborns are not routinely screened for congenital CMV (cCMV) (4, 5); as a consequence, many infants experience missed or delayed diagnoses, and treatment is frequently not initiated within the recommended timeframe of 1 month of life (6).

### cCMV prevalence

**Figure 1:**
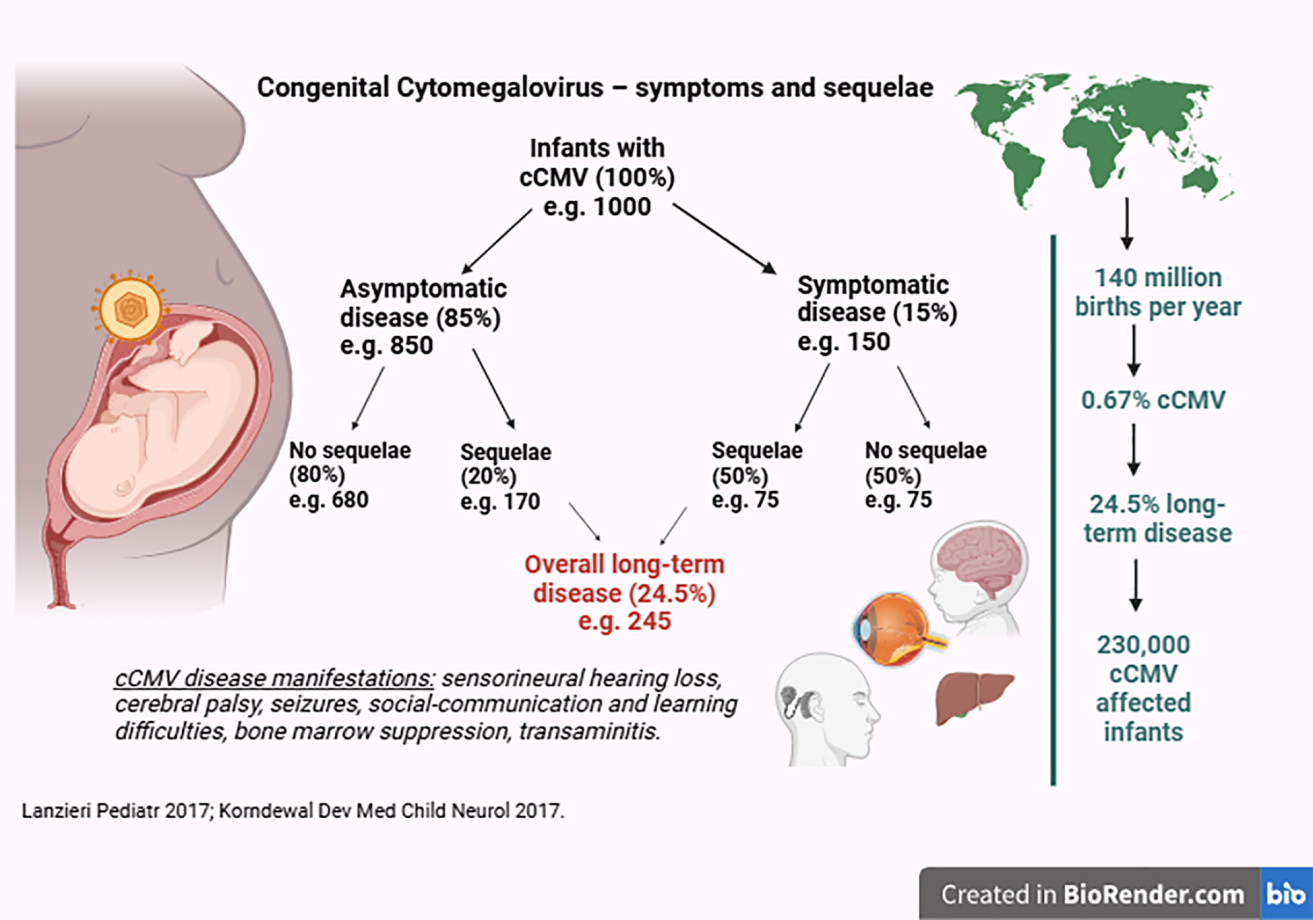
Congenital cytomegalovirus—proportion of infants with symptoms and sequelae (created in BioRender).

cCMV affects approximately 1 million infants globally each year, accounting for 0.67% of all births (7). In high-income countries, the median prevalence is 0.48% (CI 0.40%–0.59%) (7). cCMV contributes to around 10% of childhood cerebral palsy (8) and 25% of sensorineural hearing loss (SNHL) (9) and is increasingly recognized to be linked to sociobehavioural, communication, and learning difficulties (10–13). One of the major barriers to universal screening is that while an estimated 25% of infants with cCMV develop long-term significant sequelae, the rest do not (Figure 1) (4, 5, 11); moreover, these sequelae may not be present in the neonatal period. SNHL can have a late onset in 11.5% of cases (14, 15), and neurodevelopmental outcomes can be difficult to predict.

### cCMV clinical presentation

Infants are classified as symptomatic or asymptomatic, with symptomatic infants most frequently defined as those with abnormalities detected on physical examination, basic blood tests, ophthalmologic evaluation, and audiologic assessment (16). Up to 15% of babies with cCMV show symptoms at birth (4, 5); however, unless an affected infant presents with severe manifestations of cCMV disease, the signs and symptoms can be subtle and often go unnoticed, resulting in fewer than 25% of these children being diagnosed within the first month of life (6). Features of intrauterine growth restriction (IUGR), petechiae, anaemia, hepatosplenomegaly, jaundice, and microcephaly do not always appear together and can be caused by other aetiologies. This is compounded by a genuine issue of lack of awareness among relevant healthcare professionals: obstetricians, midwives, neonatologists, paediatricians, and audiologists, with awareness reported to be as low as 23% (17, 18). Among infants classified as asymptomatic, up to 20% develop late-onset SNHL, developmental delays, and social communication or learning difficulties, and these infants often remain undiagnosed until after 1 year of age (10, 19, 20).

### cCMV diagnosis

cCMV is diagnosed by identification of CMV in urine, saliva, or blood via polymerase chain reaction (PCR) within the first 21 days of life. Beyond 21 days, CMV identified may be due to postnatal CMV, typically due to breast milk transmission. It is critical to differentiate between postnatal and congenital CMV because postnatal CMV in term immunocompetent infants usually does not cause disease.

Studies on infants with cCMV in the first few weeks to months of life have shown reduced effector cytokine responses and markedly lower polyfunctionality of newborn CD4 and CD8 T cells (21, 22). These data infer a reduced ability to control CMV, thereby reinforcing the importance of early initiation of antiviral therapy.

### cCMV treatment

Treatment for cCMV involves twice-daily oral valganciclovir for 6 months, although a 6-week course may now be considered sufficient for mildly affected infants (16). However, side effects, including neutropenia or transaminitis, can occur in 20% of infants (2, 3), requiring blood test monitoring throughout their treatment on at least a monthly basis. While animal data suggest a theoretical risk of oncogenesis and infertility secondary to valganciclovir, no such effects have been seen in humans, although 6-month treatment regimens have only been commenced after 2015 (3). In view of these potential side effects, decisions to start treatment need to be pragmatic and evidence-based, supported by international guidelines (16).

### Targeted testing for cCMV

Targeted testing for cCMV in infants with confirmed hearing loss is increasingly performed by audiologists (23, 24) using a saliva swab sent for CMV PCR analysis; however, treatment initiation is still not timely because many infants are diagnosed with CMV after 21 days of age (25). It will then take a further 2–4 weeks before being seen by paediatric infectious diseases specialists, investigated accordingly, and started on treatment if required (6), resulting in considerable delays in initiating treatment. In some areas, newborn hearing screeners are testing for CMV after the first failed hearing test and referring directly to paediatrics thereafter, although this is not a routine national practice (27).

### Potential for universal screening for cCMV

There are two potential universal screening approaches: antenatal maternal serological screening in early pregnancy and neonatal screening after birth. A few European countries perform CMV serology in early pregnancy since significant long-term sequelae are limited to first-trimester infections, and there is an effective maternal antiviral treatment to prevent CMV transmission to the foetus (27). The main limitations of this approach are that CMV serology should be performed very early in pregnancy (i.e., 6–8 weeks) (16) and can only identify primary infections, which may reflect less than half of CMV infections during pregnancy in European settings (28).

Infant dried blood spot (DBS) samples collected on day 5 of life in the United Kingdom are used to screen for diseases such as cystic fibrosis, sickle cell anaemia, hypothyroidism, severe combined immunodeficiency, and certain rare metabolic disorders. The DBS presents an opportunity for early screening of cCMV, facilitating timely treatment initiation. Many of the standardised criteria required for universal screening have already been reached for cCMV e.g., (1) the condition is significant in both frequency and severity; (2) it often presents with mild, pre-symptomatic, or asymptomatic manifestations that might not be otherwise identified; (3) there is an agreed policy for further investigation (16); and (4) cost-effective treatment interventions supported by RCT are available (2, 3) and, importantly, should be commenced early, at least before 1 month of age. The use of the DBS as a screening tool is largely acceptable to the general population and has recently been used in universal screening programmes for cCMV in the United States and Canada (29–31).

The reasons given by the UK National Screening Committee (UK-NSC) for not providing screening for cCMV are: (a) the uncertainty regarding whether screening tests can identify infants with cCMV who will develop long-term neurodevelopmental and hearing problems; (b) lack of evidence that early treatment following screening leads to better outcomes than later treatment after symptoms appear; (c) uncertainty over whether asymptomatic infants require treatment; and (d) concern regarding the identification of a large number of infants with cCMV, leading to further investigations, parental anxiety, and cost to the NHS (32).

### Addressing the UK-NSC concerns raised against universal screening for cCMV

#### Can screening tests identify infants with cCMV who will develop long-term sequelae?

Yes. The use of DBS for cCMV screening has a relatively high analytical sensitivity of 85.7%, along with 100% specificity and a 98% positive predictive value (33) compared to saliva testing, thereby identifying the majority of cCMV cases and those more likely to develop sequelae. Up to 20% of asymptomatic infants develop long-term sequelae, amounting to 69% (170/245, Figure 1) of all infants with cCMV. Universal screening would enable identifying these infants who might otherwise be missed. Notably, recent experience in Canada demonstrated that most symptomatic cases would have been missed without universal screening (31). After case identification, a paediatrician would be required to determine whether these infants are at risk of longterm disease. This evaluation would include a physical examination, full blood count, alanine transaminase measurement, cranial ultrasound, audiology, and ophthalmology performed to detect any signs of disease (34).

Long-term sequelae from cCMV arise predominantly due to CMV transmission during the first trimester of pregnancy (35, 36), which is the primary phase of embryological development and immunological immaturity. Increasingly, cCMV research focuses on ways to identify first-trimester infections and predict infants at risk of long-term disease, equipping clinicians with the necessary information to guide treatment initiation (37). Infants with cranial ultrasound abnormalities undergo brain magnetic resonance imaging (MRI), with findings assessed using a cCMV-specific MRI brain score (38–40). This score aligns with physical outcomes (41) and is being validated for functional development in ongoing studies. Additional biomarker research includes gene signatures predictive of late-onset SNHL (42), reduced or absent CMV-specific immune responses (21, 43), markers of bone marrow function (44), and virological factors (45–47).

#### Does early treatment lead to better outcomes?

RCTs have demonstrated reduced SNHL and improved developmental outcomes in symptomatic infants, particularly those with central nervous system (CNS) disease, after initiating treatment within 28 days of life (2, 3). Subsequent non-randomised studies suggest that starting treatment up to 2 months of age may be similarly effective, but these studies had only 6 months of follow-up (48). A recent RCT initiating valganciclovir treatment in children aged between 4 weeks and 4 years of age did not demonstrate benefit from a 6-week course despite 70% of participants having CNS disease (49). In contrast, the Concert study, although not an RCT, demonstrated improvement in hearing but not development at 20 months after 6 weeks of treatment with valganciclovir in infants with isolated SNHL aged up to 12 weeks (50). Overall, these findings suggest that for infants with more severe CNS disease, earlier treatment remains critical, aligning with their limited inability to control CMV in the neonatal period. In contrast, infants with isolated SNHL appear to benefit from treatment even when started up to 12 weeks of age (50). The benefit of universal screening for these infants is easier differentiation between congenital and postnatal CMV, the latter of which does not require treatment in term infants.

#### Do asymptomatic infants require treatment?

A European consensus of experts does not recommend treating infants with isolated IUGR without other manifestations of cCMV at birth (16). Due to poor recruitment, RCTs have not been able to address the treatment of infants considered asymptomatic (51–53). However, two observational studies have demonstrated abnormal brain MRI findings in almost half of the infants considered asymptomatic, either picked up through targeted screening or research screening programmes (54, 55). A retrospective study demonstrated that up to 18% of children with asymptomatic untreated cCMV exhibited neurocognitive abnormalities at 6 years of age, which was significantly higher than CMV-uninfected children (10). These studies suggest that the long-term impact of cCMV may be greater than previously recognised, and this raises the question of whether asymptomatic infants with evidence of CNS disease should be considered for treatment. Historically, asymptomatic infants have included those with SNHL, and as mentioned above, the Concert study has suggested that treatment could be beneficial for this population (50).

#### Would universal screening result in unjustified parental anxiety and cost to the NHS?

As universal screening programmes are underway in the United States and Canada, it has become evident that concerns regarding parental anxiety should not be a deterrent. Parental attitudes have been overwhelmingly supportive and in fact revealed frustration over the lack of public, particularly pregnant women's awareness of cCMV and the possibility of prevention (56). The benefits of newborn screening (NBS) for cCMV would be experienced by the affected infant receiving timely diagnosis and treatment, maximising their potential development during early childhood. It would also be experienced by the affected families through the reduction in the significant psychological impact of the “diagnostic odyssey” that has frequently been described (57, 58). Screening for cCMV is not only ethical and rightly beneficial for affected infants and families but also financially prudent, as cases of cCMV are estimated to cost the UK economy £750 million pounds annually, encompassing both NHS expenses and societal costs (57, 58); the cost-effectiveness of screening for cCMV has been proposed (59).

Collectively, these data have begun to address the concerns raised by the UK-NSC, and there is growing recognition of the pressing need to improve the care provided to infants with cCMV. As barriers to screening are addressed and universal screening strategies are increasingly being embraced worldwide, appropriate screening tools must be made available to facilitate accurate diagnostics. Our study aimed to demonstrate the use of two potential screening approaches, dried blood spots and dried saliva spots, for the timely identification of infants with cCMV.

## Methods

### CMV quantitative PCR assay development

A dilution series was prepared using a CMV viral preparate (WHO International Standard for Human Cytomegalovirus for Nucleic Acid Amplification Techniques, NIBSC) containing 0.68 (CMV1), 2.04 (CMV2), 3.4 (CMV3), 6.8 (CMV4), and 340 (CMV5) IU/reaction. A sample without the CMV preparate was used as a control (CMV0). A research use-only commercial kit was developed to identify CMV in infant DBS samples by targeting a conserved region of CMV DNA and the RRP30 control gene (Eonis CMV qPCR kit, Revvity Inc.). The dry chemistry real-time qualitative PCR (qPCR) kit contains the reagents needed to assess the cCMV status by qPCR, utilising a simple one-step DNA elution process. To each well, 100 µL of elution solution (Revvity Inc.) was added; the plates were sealed with adhesive foil seals and incubated at 70°C for 20 min while being agitated at 700 rpm to elute DNA from the DBS samples. Afterward, the plates were cooled at room temperature for 10 min and briefly centrifuged. Next, 20 µL of eluate was transferred to dry chemistry qPCR plates containing the dried qPCR reagents (Revvity Inc.), and the plates were sealed with an optical PCR seal. The plates were vortex-mixed, briefly centrifuged, and then subjected to qPCR using a qTower qPCR instrument (Analytic Jena) under the following conditions: 95°C for 3 min, 45 cycles of 95°C for 10 s and 61°C for 1 min, with fluorescence measurement for FAM (control gene RRP30) and ROX (CMV). Post-run analysis was performed using LEDTest 96 software (version 1.0).

### Study populations

This study was approved by the Health Research Authority (IRAS: 296570) and the Research Ethics Committee (REC: 21/EM/0241), and the assay was tested on three population groups: (1) DBS samples taken on Guthrie cards were collected as part of the routine NBS programme in the United Kingdom, and leftover DBS material was used for cCMV testing; (2) leftover blood from children known to have CMV viraemia (not due to congenital infection) was applied to DBS cards and used as positive controls; and (3) blood and saliva samples were collected from newborn infants with and without cCMV as part of an affiliated study and applied to DBS cards (IRAS: 302667, REC: 22/PR/0895).

#### Population 1: leftover DBS samples from Guthrie cards

Following routine screening on Guthrie cards collected between September 2022 and March 2023, DBS samples were stored at room temperature with a desiccant for up to 10 days to allow for the reporting of routine NBS results. The DBS samples were then punched and used in the assay. These DBS samples were obtained from the Great Ormond Street Hospital (GOSH) NBS laboratory, which serves Northeast London, most of Essex and Hertfordshire, and some of Bedfordshire and Kent and reflects a highly diverse ethnic population.

#### Population 2: leftover blood from children known to have CMV viraemia

Children aged up to 5 years with suspected CMV infection (not due to congenital CMV) were tested following standard practice at the routine clinical laboratory at GOSH (60). DNA was extracted from ethylenediaminetetraacetic acid (EDTA) blood samples, and CMV DNA levels were measured using qPCR (61), with levels reported as IU/mL. Fifty microlitres of leftover EDTA blood from these children was applied to standard Whatman 903 DBS cards to prepare CMV-positive DBS samples. These samples were dried at room temperature for at least 24 h prior to testing. Once prepared, these DBS samples were stored at −20°C with a desiccant.

#### Population 3: infants with and without cCMV

cCMV was confirmed in infants based on at least two positive samples from blood, urine, or saliva obtained before 21 days of age. These infants were identified due to symptomatic disease or failed newborn hearing screening. Infants without cCMV, aged up to 3 months, were recruited from outpatient clinics while undergoing routine blood tests for non-infectious conditions, e.g., renal anomalies, prolonged jaundice, etc. Control infants were confirmed to be CMV-uninfected, with no CMV detectable in both blood and saliva samples. Blood was collected by venepuncture in EDTA tubes, and within 24 h of collection, 50 µL was applied to DBS cards. Saliva samples were collected using Sigma Virocult cotton swabs, approved for clinical use, placed in viral transport media, and then applied to the DBS card within 24 h. Both saliva and blood DBS cards were frozen at −20°C with a desiccant. All samples were anonymised, and the laboratory and analysis team were blinded to whether the samples belonged to cases or controls.

### DBS preparation, DNA elution, and qPCR

The research use-only commercial kit, as described above, was used to identify CMV in infant DBS samples and dried saliva samples from the three populations. A 3.2-mm punch was taken from each DBS and transferred into individual wells of a 96-well plate alongside a negative and positive control provided by Revvity Inc. DNA was eluted using a one-step process in 100 µL, and 20 µL of the elute was transferred to dry chemistry qPCR plates containing the dried qPCR reagents (Revvity Inc.); the plates were sealed with adhesive foil, incubated at 70°C for 20 min, agitated at 700 rpm, cooled at room temperature for 10 min, briefly centrifuged, and then subjected to qPCR, as described previously. The experiment was performed as three individual assays using different blood donors and differing sample replicate numbers.

## Results

### Analytical sensitivity

The analytical sensitivity was determined using five samples with varying CMV levels from crude blood extracts and to be 2.04 CMV IU/reaction, equivalent to 3,000 CMV IU/mL of blood. This was the lowest sample level, with over 95% of replicates detected positive for CMV and a low Ct-value standard deviation within sample groups (Table 1). RPP30 was successfully amplified from every sample replicate.

**Table 1:** Analytical sensitivity in crude blood extract samples.

Sample	CMV IU/reaction	CMV				RPP30			
		Mean Ct	SD Ct	Replicates	CMV detected (%)	Mean Ct	SD Ct	Replicates	RPP detected (%)
CMV0	0.00	No ct	0.00	36	0	28.72	0.71	36	100
CMV1	0.68	39.92	1.01	19	74	26.35	1.61	19	100
CMV2	2.04	37.8	1.18	31	97	27.24	1.68	31	100
CMV3	3.40	37.49	0.79	31	100	27.37	2.00	31	100
CMV4	6.80	36.36	0.66	19	100	26.02	1.25	19	100
CMV5	340	30.48	0.21	19	100	25.23	0.39	19	100

Crude blood extract samples negative for CMV (CMV0) or containing various amounts of CMV preparate (CMV1-5) were analysed to determine the analytical sensitivity of the Eonis CMV qPCR kit (Revvity Inc.). The presented data are a compilation of three individual experiments with varying sample replicates. The mean and standard deviation (SD) of RRP30 and CMV cycle threshold (Ct) are given for each replicate. The percentage of each CMV and RPP30-positive replicate with detectable CMV and RPP are provided.

### Internal quality control

For internal quality control, as provided by Revvity Inc., the expectation was to observe the positive control to show the amplification for both the housekeeping gene Ribonuclease P protein subunit p30 (RPP30) and CMV. The negative control was expected to show amplification for RPP30 but not CMV. All positive kit controls show successful amplification for RPP30 and CMV, and the negative kit controls show amplification for RPP30 but not CMV. The average cycle threshold (Ct) values for the positive kit controls were 27.38 for RPP30 (range 23.90–30.77, standard deviation 1.71) and 29.35 for CMV (range 28.34–30.78, standard deviation 0.55).

### Population 1: leftover DBS samples from Guthrie cards

**Figure 2:**
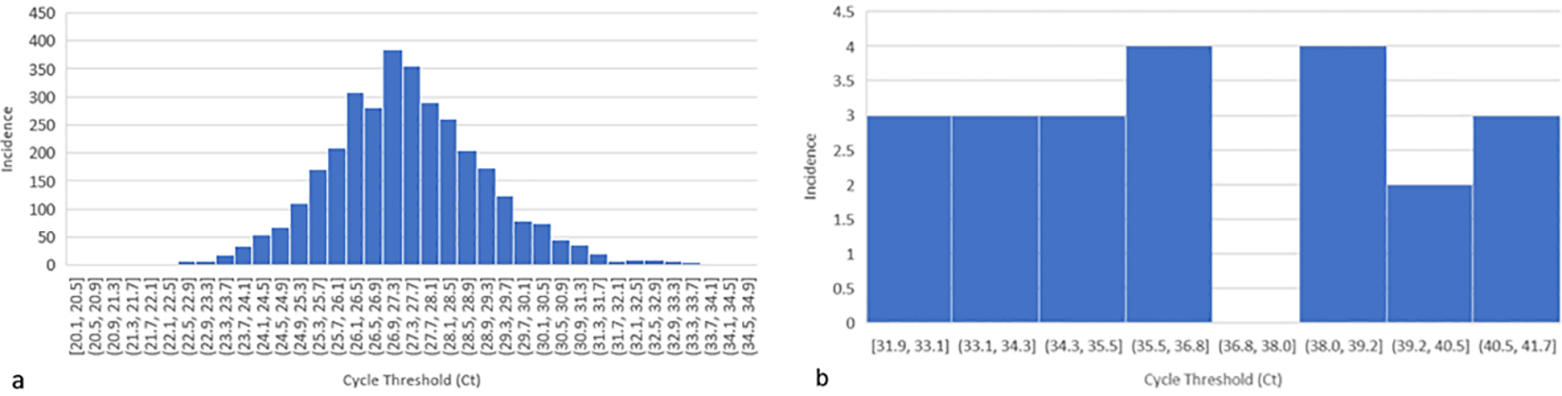
Frequency of DBS results for RPP30, housekeeping gene **(a)** and CMV **(b)**. **(a)** Histogram showing the normally distributed data for the 3,345 anonymised DBS samples. RRP30 Ct values (*x*-axis) and incidence of these Ct values (*y*-axis). The cycle threshold (Ct) value represents the number of PCR cycles required before the amplification of the target. RPP30 is the housekeeping gene, which demonstrates that the DBS punching and DNA elution have been successful. **(b)** Histogram showing the distribution of positive CMV Ct values for 22 anonymised DBS (*x*-axis) and incidence of these Ct values (*y*-axis).

In total, 3,345 anonymised DBS samples from newborn infants in the United Kingdom were obtained and analysed. No clinical data were available for these infants. All samples showed successful amplification for the RPP30 control gene. The mean cycle threshold (Ct) value for RRP30, representing the number of PCR cycles required before amplification of the target, was 27.41 (range 20.10–34.63), showing a normal distribution (Figure 2a) across all runs. Of the 3,345 anonymised unknown samples, 22 exhibited detectable CMV levels (0.66%, Figure 2b). The mean Ct value for CMV was 36.70 (range 31.87–41.68).

### Population 2: leftover blood from children known to have CMV viraemia

Ten known CMV-positive EDTA blood samples were used to create DBS samples with varying CMV levels (viral load range 1,228–640,145 IU/mL, Table 1). No other data were available for these samples, and they were added randomly throughout the various PCR runs. All samples showed successful RPP30 amplification. CMV was detected in all replicates for 7 of the 10 samples, with the lower limit for reliable CMV detection being 10 CMV IU/reaction while no detection was observed above 1.27 IU/reaction (Table 2). These findings are consistent with assay development results, which demonstrated a sensitivity limit of 2.04 CMV IU/reaction. The remaining three samples either showed no amplification in any replicate (P3) or only partial amplification in some of the replicates (P1 and P2), and these samples had the lowest CMV levels, implying that the reliable limit of CMV detection of this test is likely between 2,000 and 14,000 IU/mL; e.g., a sample with 1,872 IU showed no detectable CMV with this test, while a sample with 14,834 IU was reliably detected with a Ct value of 35.89.

**Table 2:** Details of the laboratory-created CMV-positive DBS samples.

Control	Viral load IU/mL	IU/reaction	RRP30		CMV		Replicates run	CMV detected (%)
			Mean	SD	Mean	SD		
P8	6,40,145	435.30	27.51	N/A	33.15	N/A	1	100
P7	1,28,514	87.39	25.65	0.66	32.68	1.19	2	100
P6	1,22,407	83.24	24.90	1.93	35.49	1.17	5	100
P9	77,317	52.58	26.03	N/A	32.07	N/A	1	100
P10	38,462	26.15	25.18	N/A	31.84	N/A	1	100
P5	14,962	10.17	25.10	1.92	34.16	2.18	7	100
P4	14,834	10.09	25.94	0.69	35.89	1.55	5	100
P3	1,872	1.27	23.38	0.48	0	N/A	2	0
P2	1,690	1.15	24.59	0.42	41.10	5.49	2	50
P1	1,228	0.84	25.88	0.95	35.86	0.57	6	33

Each DBS was run in a single well within a plate, and where sample volume permitted, more than one replicate was run in separate plates for each DBS. The mean and standard deviation (SD) of RRP30 and CMV cycle threshold (Ct) are given for replicates of each DBS. The percentage of each CMV positive replicate with detectable CMV is provided.

### Population 3: infants with and without cCMV

The median age was 56 (IQR 45.5–72.5) days for cases and 44.5 (IQR 6.25–83.75) days for controls (Table 3). There were no significant differences in gestational age at birth, sample age, and head circumference; however, infants with cCMV had a lower birthweight, and controls were more likely to be boys. Among infants with cCMV, 71% (5/7) had faetal abnormalities, 57% (4/7) had IUGR, 43% (3/7) had SNHL, and 57% (4/7) had an abnormal brain MRI. Bone marrow and liver function tests were normal. Two infants were known to have a primary first-trimester infection, three had a reinfection or reactivation, and in two infants, the type of congenital infection was unknown. At the time of DBS and saliva sampling for this study, four infants (57%) were on treatment, and five (71%) had detectable CMV viraemia.

**Table 3:** Characteristics of infants with and without cCMV.

Characteristics	cCMV cases, *n* = 7 Med [IQR], *n* (%)	CMV-uninfected controls, *n* = 4 Med [IQR], *n* (%)	*p*-Value
Gestational age (weeks)	37 [37–39]	39.9 [39.1–40.5]	0.06
Male	4 (57%)	4 (100%)	<0.0001
Birth weight, grams	2,950 [2,300–3,025]	3,687 [3,653–3,727]	<0.001
Head circumference (cm)	33.6 [31.5–36.5]	36 [35.3–36]	0.20
Age at sample (days)	56 [45.5–72.5]	44.5 [6.25–83.75]	0.33
Diagnosis age (days)	3 [1–19.5]	NA	NA
Age started treatment (days)	31 [12.5–43.5]	NA	NA
On treatment at sample	4 (57%)	NA	NA
CMV viraemia at sample	5 (71%)	NA	NA

“At sample” refers to the time at which the DBS and saliva samples were taken. Where applicable, characteristics were compared using an unpaired *t*-test.

All DBS and saliva samples from seven infant cases and four controls showed successful RPP30 amplification, with a mean Ct of 29.49 (range 24.41–31.58) for DBS and a mean Ct of 29.49 for saliva (range 25.57–31.57; Table 4). Only one of seven of the DBS samples from infants with cCMV detected CMV, with a Ct value of 37.16, and this DBS sample belonged to an untreated infant with the highest CMV blood viral load of 2,340 IU/mL. This result is consistent with the limit of detection in assay development (Table 1) and the findings from laboratory-created CMV-positive DBS samples (Table 2). Two infants had undetectable CMV viraemia, and three infants had CMV levels below than the detection threshold of the assay. The one infant detected (B5, Table 4) had 1.64 CMV IU/reaction, which was below the lower limit suggested by the assay development experiments.

**Table 4:** Dried blood spots and dried saliva spots taken from infants confirmed to have cCMV.

Study ID	Control or case	Dried blood spot				Dried saliva spot				On treatment	Known CMV viraemia IU/ml	CMV IU per reaction
		Ct		Detected		Ct		Detected				
		RPP30	CMV	RPP30	CMV	RPP30	CMV	RPP30	CMV			
B1	Control	27.37	No Ct	POS	NEG	30.86	No Ct	POS	NEG	NA	NA	NA
B2	Control	31.4	No Ct	POS	NEG	30.37	No Ct	POS	NEG	NA	NA	NA
B3	Control	24.41	No Ct	POS	NEG	31.27	No Ct	POS	NEG	NA	NA	NA
B4	Control	31.58	No Ct	POS	NEG	31.57	No Ct	POS	NEG	NA	NA	NA
B5	Case	29.52	No Ct	POS	NEG	29.17	19.83	POS	POS	No	Yes 2094	1.46
B6	Case	26.98	No Ct	POS	NEG	25.57	27.4	POS	POS	Yes	No <242	NA
B7	Case	27.98	37.16	POS	POS	29.6	30.97	POS	POS	No	Yes 2340	1.64
B8	Case	25.61	No Ct	POS	NEG	28.82	27.6	POS	POS	No	Yes 444	0.31
B9	Case	28.37	No Ct	POS	NEG	28.75	32.81	POS	POS	Yes	Yes 80	0.06
B10	Case	30.14	No Ct	POS	NEG	29.85	33.02	POS	POS	Yes	No <34.5	NA
B11	Case	29.06	No Ct	POS	NEG	28.54	35.01	POS	POS	Yes	72	0.05

Cycle threshold (Ct) value, represents the number of PCR cycles required before amplification of the target. RPP30 is the house-keeping gene which allows for quantification of the DNA present in each reaction. RPP30 or CMV detected is represented by “POS”, and RPP30 or CMV not detected is represented by “NEG“. CMV viraemia was performed in NHS laboratories as per clinical care, measured in international units per ml blood. The limitation of the NHS CMV PCR assay varies between runs hence the lowest detectable level is not consistent. CMV IU / reaction reflects how many IU of CMV were in the DNA PCR reaction well based on the volume of blood applied to the DBS (50µl) and the size of the DBS punch (2.3mm diameter), therefore equivalent to 3.5µl blood, 20% of which was used for qPCR.

All seven saliva samples showed positive CMV amplification, with a mean Ct of 29.52 (range 19.83–35.01) for CMV-positive saliva samples (Table 3), regardless of blood viraemia and treatment status. All CMV-uninfected infants were confirmed to have no CMV detectable in their saliva.

## Discussion

This is the first retrospective study to examine the use of DBS to determine the incidence of cCMV in the United Kingdom, and it represents the largest prevalence study since 1983 (62). The UK prevalence of cCMV has not been reported since 1991, when it was estimated as 0.33% (95% CI 0.15–0.62) (63). However, our study demonstrates a potential cCMV rate of 0.66% among the examined infant population using the high-sensitivity assay on DBS. With the current rate of annual births of approximately 600,000 in the United Kingdom (64) the rate of 0.66% may reflect 3,960 infants with cCMV each year, a quarter of whom may develop long-term sequelae.

The assay offers a single-step DNA elution and processing approach that efficiently streamlines CMV PCR analysis for NBS. The cCMV NBS assay is a DNA-based assay as per the widely adopted SCID and SMA NBS tests; however, the cCMV assay used here is dry chemistry-based, reducing hands-on time, and requires only DNA extraction from the DBS, already done in many labs, followed by a manual transfer to the qPCR plate. The approximate hands-on time for running one 96-well plate after DBS punching is 30 min, with the whole process taking around 3 h. Therefore, overall costs, including staff labour, are expected to be less than those of SCID or SMA NBS.

The analytical sensitivity of the assay is notable at 2.04 CMV IU/reaction. In addition, the low Ct-value standard deviation demonstrates high sample uniformity and assay repeatability in crude blood extract samples. The criteria used in this study were that any amplification of CMV would be considered a positive result, regardless of Ct value. However, without verification using a separate clinical sample, such as urine or saliva, we cannot be certain that this reflects the true prevalence rate. No clinical data were available for the infants whose DBS samples were analysed; therefore, it was not feasible within the design of this study to verify the cCMV diagnosis and clinical outcomes. By running a prospective study with sample verification, it would be possible to determine the precise cut-off level needed for cCMV diagnosis that is relevant for clinical care.

We attempted to verify the sensitivity of the DBS samples by testing 17 infant/child samples with known CMV viral loads confirmed in routine clinical laboratories, ranging from 34.5 to 640,145 IU/mL. The NBS assay reliably detected CMV at levels down to 2,340 IU/mL. CMV levels below this threshold were not detected in every replicate due to a dilutional effect and differing quantities of CMV estimated per PCR reaction; however, it was evident that detection was possible even at 1.64 CMV IU/reaction. Thus, the sensitivity limit appears to be between 2,000–3,000 CMV IU/mL. This is lower than the reported sensitivity of 500 IU/mL achieved by nested PCR and currently in use by Health Service Laboratories in London (65), where retrieved DBS samples are tested after a clinician’s request in an attempt to confirm cCMV diagnosis in infants presenting beyond 21 days of age.

Ct values above 37.16 were less reliably reproduced in known viral load titrations. These were blood samples from children up to 5 years old and processed in real time, without a corresponding storage period. It is possible that all CMV-positive cases in the anonymised DBS samples tested were true CMV positives, suggesting that the incidence of cCMV in the population is now higher than previously estimated. Of the DBS samples obtained from the NBS laboratory, nine had a Ct value of 37.16 or higher, so an alternative possibility is these might represent false positives. If we use 37.16 as the cut-off value to reliably determine CMV-positive from CMV-negative cases, this adjusts the cCMV incidence of our population to 0.39%, aligning more closely with the incidence estimates of the 1991 study (60). Other new NBS tests introduced into health systems often review cut-offs and algorithms as large population datasets are uncovered [e.g., for the SCID NBS assay (66)]. However, since the purpose of this assay is screening rather than diagnosis, we suggest using a generous cut-off to avoid missing low-level cCMV cases in newborns. Diagnosis could then be confirmed using CMV PCR from urine or saliva without incurring significant costs.

CMV was identified from the DBS in one of the seven infants with confirmed cCMV, five of which had CMV viraemia. The assay detected CMV in the infant with the highest viral load of 2,340 IU/mL but not in four infants with known CMV viraemia ranging from 72 to 2,094 IU/mL. However, all four of these infants had CMV levels below the lower limit of the assay in the PCR reaction. The median age of testing in this population was 33 days, and some infants were already under treatment. Therefore, this sample population does not perfectly reflect testing in the context of NBS, where CMV viral loads may be higher closer to birth, on day 5 when the heel-prick blood test for NBS is usually performed. This is also a very small sample set that was tested once only; therefore, these results may be influenced by replicate variability at low viral loads and reflected by existing data, suggesting a DBS sensitivity of 81–83.9% (67) for cCMV diagnosis. Importantly, all our cases needed treatment, so universal screening using DBS might have missed six of these seven infants and would have relied upon these infants being identified by other means (as was the case in reality). While the assay demonstrates high analytical sensitivity, maximising the clinical sensitivity of CMV detection from DBS in NBS may need different DNA extraction protocols, e.g., purification methods allowing more DNA molecules in the reaction through concentration of target analytes or utilisation of two DBS punches would be practical and feasible.

In contrast, all saliva samples from infants with confirmed cCMV accurately detected CMV to a maximum Ct value of 35.01, and correspondingly, none of the saliva samples from the controls showed CMV. This implies that saliva may be the preferable sample type for cCMV testing compared to blood and aligns with literature that recognises even high-sensitivity DBS assays can identify only up to 81% of infants since not all infants with cCMV exhibit CMV viraemia at diagnosis (67). It was previously thought that viraemic infants may be at a higher risk of developing symptoms and more severe SNHL (68, 69). However, recent data using saliva-based screening approaches illustrate that 46% of asymptomatic infants had abnormal brain imaging (54), including two aviraemic cases. These infants would not have been identified by other means and would have missed the opportunity for treatment.

Saliva is known to have higher viral loads, which makes it more suitable for screening. One caveat is the limitation that saliva must be taken at least 1 h after breastfeeding to prevent breast milk contamination and false positive results (70). Although using saliva on a Guthrie card would require changes in collection practice, we demonstrate that applying saliva to a DBS card is a simple, effective, and potentially highly sensitive method for universal cCMV screening. In addition, parental acceptability of saliva-based CMV screening after a failed hearing test has already been demonstrated in the United Kingdom (71, 72).

A large-scale Israeli study demonstrated the feasibility, efficiency, and cost-sparing approach of pooled saliva testing for CMV identification, with an insignificant loss of sensitivity (73). Pooled saliva from DBS cards using this high throughput commercial assay presents a potentially cost-effective approach for CMV NBS. Further work is required to verify the use of dried saliva spots and assess the absolute sensitivity of this commercial assay in saliva, alongside a UK-equivalent cost-effectiveness analysis. Although cCMV had been confirmed previously, our study used clinical samples from infants over 21 days old. Future work should verify this assay using samples from 5-day-old infants when the Guthrie test is performed. Thereafter, a pilot study could be conducted to confirm practical feasibility, parental acceptability, and no loss of sensitivity when pooling saliva from DBS cards.

Our study demonstrates a high-sensitivity commercial assay for the detection of cCMV to be used alongside the NBS heel-prick blood test performed at 5 days of age. Additionally, dried saliva spots to test for CMV showed even higher sensitivity with this assay, presenting a potentially cost-effective approach in the context of pooled testing. Using either approach would help address the enormous unmet clinical need for the timely identification of cCMV in the neonatal period.

Analysis of more than 3,345 stored newborn DBS samples revealed a potential cCMV prevalence of 0.66% in the United Kingdom. Parallel clinical analysis of these infants is required to ascertain whether the diagnosis of cCMV could be confirmed in urine or saliva and whether these infants have cCMV disease that requires treatment. However, this could reflect up to 1,000 infants each year in the United Kingdom who need early treatment that could improve their health outcomes. Considering this significant morbidity burden, that research is increasingly addressing the previous concerns of the UK-NSC, and that both antenatal and neonatal screening programmes have been demonstrated to be effective, now is most certainly the time for the United Kingdom to reconsider the feasibility of universal screening for cCMV.

## Data Availability

The raw data supporting the conclusions of this article will be made available by the authors without undue reservation.
